# Dr. Charles C. Chittenden

**Published:** 1905-12

**Authors:** 


### DR. CHARLES C. CHITTENDEN.

Dr. Charles C. Chittenden, of Madison, Wis., died Friday evening, December 15, 1905.

This brief announcement, coming too late for extended notice, brought to us, as it will bring to Dr. Chittenden's friends throughout the country, a sense of unexpected loss, for it is probable few were aware of his extreme illness leading to no hope of final recovery.

His name in the dental profession was a synonym for all that stood for progress. Whenever it was deemed necessary to advance the educational standard there was always to be found Dr. Chittenden. While his work has been national, his principal effort has been in Wisconsin, his own state. There he has been able to mould professional sentiment to a standard higher than that of other sections. The impress of his energy and supreme devotion to the better professional life, has borne rich fruit, and though his voice and pen are silenced, his work and influence will remain as the standard for the dental profession of the United States to work up to and adopt.

Space will not permit at this time to enter into details of his life work. Our friend and co-worker has passed on to the road that all humanity must travel. May we all be equally able to prove, as he did, that our ambitions are based on that unselfish devotion he manifested, and in emulating his virtues, strive to finish the work he left unaccomplished.

